# Determinants of SARS-CoV-2 waning immunity in allogeneic hematopoietic stem cell transplant recipients

**DOI:** 10.1186/s13045-022-01250-2

**Published:** 2022-03-18

**Authors:** Mathieu Leclerc, Rabah Redjoul, Anne Le Bouter, Florence Beckerich, Christine Robin, Vincent Parinet, Cécile Pautas, Dehbia Menouche, Selwa Bouledroua, Lydia Roy, Ludovic Cabanne, Yakout Nait-Sidenas, Slim Fourati, Sébastien Maury

**Affiliations:** 1 Hematology Department, Fédération Hospitalo-Universitaire TRUE InnovaTive theRapy for immUne disordErsHenri Mondor Hospital, Assistance Publique-Hôpitaux de Paris (AP-HP), 51 avenue du Mal de Lattre de Tassigny, 94010 Créteil Cedex, France; 2 INSERM U955, Paris Est Créteil University UPEC, Créteil, France; 3 Virology Department, Henri Mondor Hospital, Assistance Publique-Hôpitaux de Paris (AP-HP), Créteil, France

**Keywords:** SARS-CoV-2, Hematopoietic stem cell transplantation, Immune response, mRNA vaccine, COVID-19

## Abstract

Hematopoietic stem cell transplant (HSCT) recipients are at high-risk for severe COVID-19 and have altered immune responses to vaccination. We sought to evaluate the dynamics of immune response to BNT162b2 mRNA vaccine in HSCT recipients. We systematically proposed vaccination with BNT162b2 to HSCT recipients and gave a third vaccine dose to those showing titers of IgG(S-RBD) below 4160 AU/mL 1 month following the second dose. We then quantified anti-SARS-CoV-2 antibodies dynamics in 133 of these HSCT recipients (88 after two and 45 after three vaccine doses) 6 months after the first vaccine dose. Mean IgG(S-RBD) titer at 6 months was significantly lower than the peak value measured 1 month after a second (*p* < 0.001) or third (*p* < 0.01) vaccine dose. IgG(S-RBD) titers at 6 months were strongly correlated to peak values (*p* < 0.001) and a peak titer above 10,370 AU/mL predicted persistent protection at 6 months. Seventy-two percent (96/133) of patients retained protective antibody levels at 6 months. Immunosuppressive drugs and low lymphocyte counts in peripheral blood correlated with lower IgG(S-RBD) titers at 6 months. Four patients (3%) developed PCR-documented SARS-CoV-2 infection and one died.

**Supplementary Information:**

The online version contains supplementary material available at 10.1186/s13045-022-01250-2.

## Introduction

Allogeneic hematopoietic stem cell transplant (HSCT) recipients are at high risk of developing severe and/or lethal forms of Coronavirus disease 19 (COVID-19) [1, 2], the entity described 2 years ago and related to the emergence in China of a novel coronavirus referred to as severe acute respiratory syndrome coronavirus 2 (SARS-CoV-2) [3, 4]. While hope has arisen a year ago from the promising results of messenger RNA (mRNA) vaccines studies [5, 6], showing high protection rates and a favorable safety profile, some concerns remain regarding the efficacy of vaccination in HSCT recipients, as humoral response might be altered in this setting because of concomitant immunosuppressive medications, and delay or alteration in B-cell reconstitution. Indeed, in a previous study focused on the vaccination of HSCT recipients with a mRNA vaccine, we reported an insufficient immune response after two doses in 41% of cases [7]. Together with the poor prognosis of COVID-19 infection in HSCT and solid-organ transplant recipients, this prompted the French National Authority of Health to recommend the use of a third dose in immunosuppressed patients poorly responding after a standard 2-doses vaccination [8]. We previously showed an improved immunogenicity of the vaccine after such a third dose in HSCT recipients [9], but the longevity of vaccine-induced immunity in this context is unknown.

## Methods

In our department, the BNT162b2 messenger RNA (mRNA) vaccine (Pfizer-BioNTech) was systematically proposed to allogeneic HSCT recipients, starting from 3 months following HSCT. One hundred and fifty eight patients received two doses given 1 month apart, while 10 other received only one vaccine dose since they had been documented with COVID-19 before vaccination. In the more recent period, 9 recipients were vaccinated with two doses before HSCT. We monitored all vaccinated patients for anti-spike glycoprotein-specific IgG [IgG(S-receptor-binding domain (RBD))] and anti-nucleocapsid protein (N) IgG titers 1 month after the last vaccine dose and gave an additional vaccine dose to recipients showing titers of IgG(S-RBD) below 4160 AU/mL at that time. Among 54 patients with IgG(S-RBD) below 4160 AU/mL after 2 vaccine doses, 47 received a third vaccine dose at 51 ± 21 days (mean ± SD) after the second dose. One patient who experienced transient facial paralysis following the second vaccine dose refused to receive a third vaccine dose despite low titer of IgG(S-RBD) following vaccination.

**Fig. 1:**
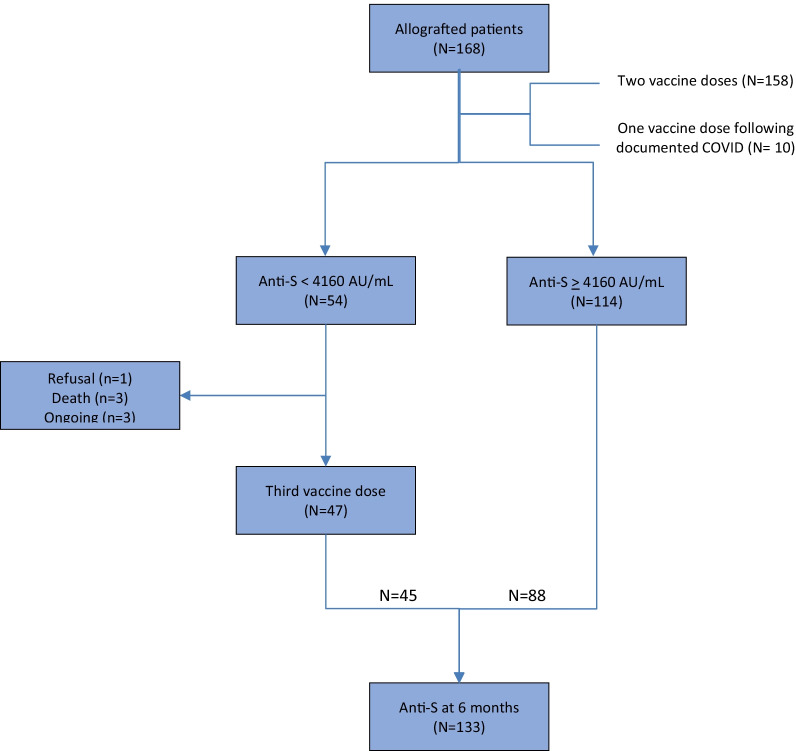
Study flowchart

As shown in study flowchart (Fig. 1), anti-SARS-CoV-2 antibodies dynamics were quantified in 133 HSCT recipients (88 after two and 45 after three vaccine doses) at 6 months following vaccination, at a mean time of 184 ± 15 days (median [IQR] 182 days [178–190]) following the first vaccine dose. All patients but two were transplanted for a hematological malignancy and 29 out of 133 (22%) were in the first year following HSCT as vaccination was initiated. We mostly observed mild to moderate side effects following vaccination (Additional file 1: Table S1). Two patients experienced transient facial palsy in the days following a second or a third vaccine dose. Both were treated with steroids with total regression in one but mild sequelae in the other.

Serum samples were analyzed for IgG anti-N SARS-CoV-2 detection (ARCHITECT®, Abbott Laboratories) and for IgG(S-RBD) IgG titration with the SARS-CoV-2 IgG Quant II assay (ARCHITECT®, Abbott Laboratories). Details regarding serological assays can be found in the Additional file 1.

Clinical and biological data were collected retrospectively from medical charts. Categorical variables were compared by Fisher exact tests. Comparisons of continuous variables means were performed using Student *t*-tests and one-way ANOVA tests, as appropriate. All tests were two sided, and the type 1 error rate was fixed at 0.05.

## Results

**Fig. 2:**
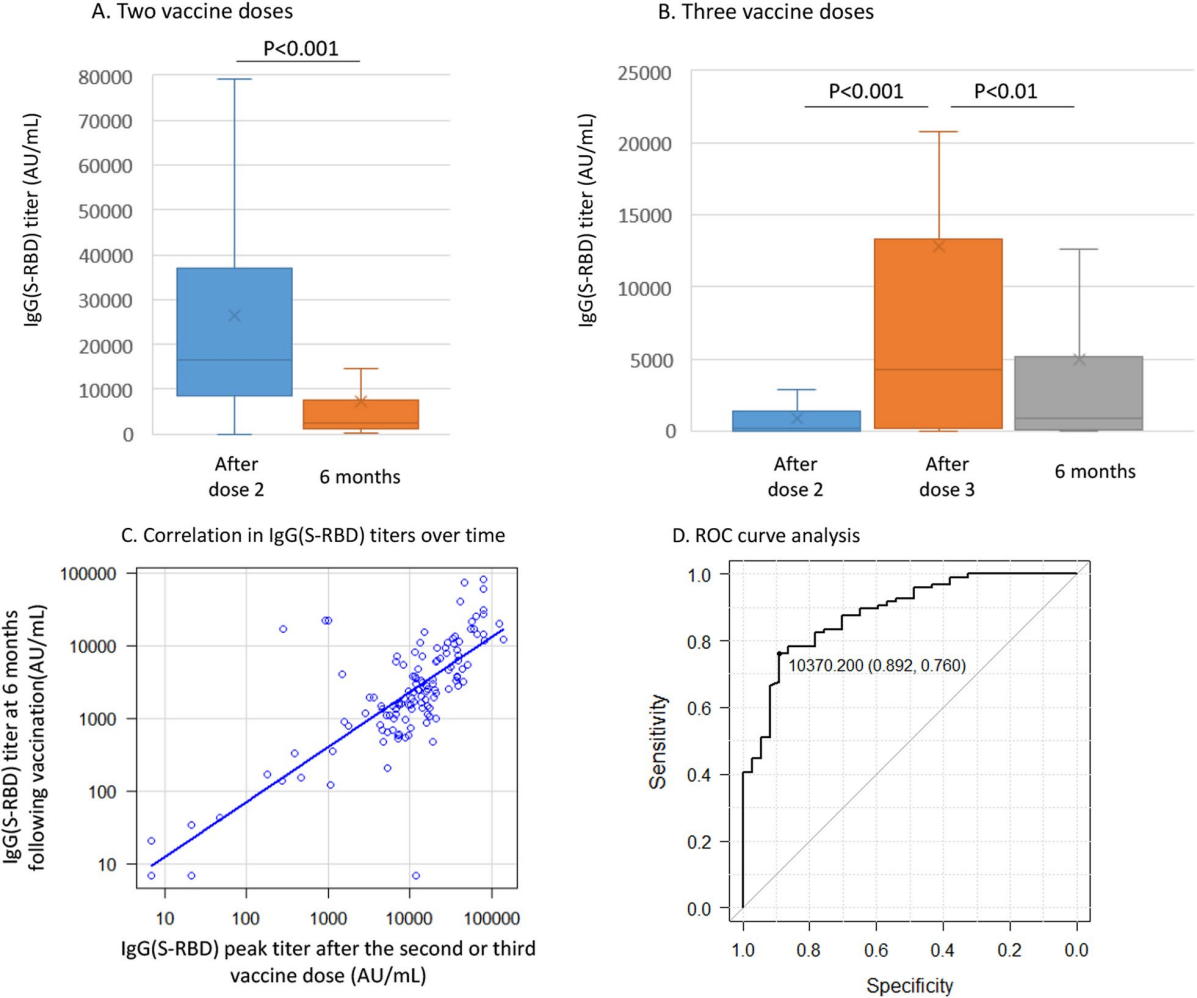
Anti-SARS-CoV-2 antibodies maintenance over time. IgG(S-RBD) were quantified after two (*n* = 88, Panel A) or three (*n* = 45, Panel B) vaccine doses (at a mean follow-up of 59 ± 17 and 103 ± 25 days following the first vaccine dose, respectively) and compared with IgG(S-RBD) quantified at 6 months (at a mean follow-up of 184 ± 15 days following the first vaccine dose). Panel C shows Spearman correlation between IgG(S-RBD) quantified (1) at the peak value after the second or third vaccine dose and (2) at 6 months after the first vaccine dose. The calculated correlation coefficient was 0.582 (95% CI 0.457–0.684, *p* = 2 × 10E−13). Panel D shows receiver operating characteristic (ROC) curve analysis for predicting IgG(S-RBD) titer above 1000 AU/mL at 6 months after vaccination. Initial IgG(S-RBD) levels were quantified after the second (*n* = 88) or, in case, the third (*n* = 45) vaccine dose. The area under curve for late humoral protection was 0.883 (95% CI 0.823–0.944)

In 45 out of 133 HSCT recipients, a third vaccine dose was given because of low humoral response after two doses (IgG(S-RBD) below 4160 AU/mL), which led to a significant increase in IgG(S-RBD) titer (12,783 ± 20,324 as compared to 839 ± 1151 AU/mL after the first 2 doses, *p* = 0.000147). Both in recipients who received two and three vaccine doses, the IgG(S-RBD) titer decreased over time with a mean titer at 6 months following vaccination significantly lower as compared to the peak value observed 1 month after the two (7064 ± 12,887 vs 26,337 ± 25,675, *p* = 6 × 10^–13^, Fig. 2A) or three vaccine doses (4957 ± 10,425 vs 12,783 ± 20,324, *p* = 0.00221, Fig. 2B). The IgG(S-RBD) titers at 6 months strongly correlated with the corresponding peak values (*p* < 0.001, Fig. 2C) and did not differ significantly, in mean values, between patients who received two or three vaccine doses (*p* = 0.331).

We chose the IgG(S-RBD) threshold above 1000 AU/mL at 6 months after vaccination to characterize a better long-term humoral protection, based on an independent study using the same Abbott Architect SARS-CoV-2 IgG Quant II assay as in the present study, showing that IgG(S-RBD) over 1000 AU /mL are able to neutralize variants of concern (including Alpha and Beta) [10]. Out of 133 recipients, 96 (72%) had level of IgG(S-RBD) above that threshold at 6 months. Given the correlation between peak IgG(S-RBD) titers and residual titers at 6 months, we tested the discrimination ability of peak IgG(S-RBD) titers for predicting residual titer above 1000 AU/mL at 6 months after vaccination, using ROC curve analysis (Fig. 2D). The area under curve (AUC) for late humoral protection was 0.883 (95% CI 0.823–0.944). The optimal cutoff of initial peak antibody titer was 10,370 AU/mL to predict an estimated protective residual IgG(S-RBD) level at 6 months.

To analyze the impact of previous SARS-CoV-2 infection on IgG(S-RBD) maintenance in vaccinated recipients, we grouped all patients showing at least one positive or limit anti-N IgG detection over sequential monitoring (Additional file 1: Table S2). As compared to patients with undetectable anti-N IgG all along follow-up (*n* = 110), previously infected HSCT recipients (*n* = 23) had higher IgG(S-RBD) titers at 6 months (14,127 ± 21,026 vs 4760 ± 8084 AU/mL, *p* = 0.00039).

We analyzed clinical and biological, patient- and transplant-related determinants correlating with a non-protective immunity at 6 months after vaccination (Table 1). In univariate analysis, the factors associated with an antibody level at 6 months below 1000 AU/mL were (1) rituximab given within the 6 months preceding vaccination (*p* < 0.001), (2) systemic immunosuppressive medication other than rituximab given within the 3 months preceding vaccination (*p* = 0.01), (3) mean lymphocyte and B-cell counts in peripheral blood (PB) over the study period below 1G/L and 0.25 G/L, respectively, (*p* < 0.01 and *p* < 0.001, respectively). Recipients who previously received a third vaccine dose because of their low humoral response after the first two doses (see inclusion criteria for a third dose), also had lower antibody levels at 6 months (*p* < 0.001). All the explicative factors identified in univariate analysis being altogether correlated, we did not run any multivariable analysis.

**Table 1:** Patient characteristics according to the obtainment of IgG(S-RBD) neutralizing level at 6 months

	IgG(S-RBD) titer at 6 months		*p* value
	< 1000 AU/mL (*n* = 37)	> 1000 AU/mL (*n* = 96)	
Male sex, *n* (%)	23 (62)	50 (52)	0.335
Recipient age at HSCT (years), mean ± SD	59 ± 11	57 ± 14	0.561
Donor age at HSCT (years), mean ± SD	38 ± 13	38 ± 14	0.769
*Disease type, n* (%)			0.906
Myeloid malignancy	28 (76)	73 (76)	
Lymphoid malignancy	9 (23)	21 (22)	
Non-malignant	0	2 (2)	
*Donor type, n* (%)			0.635
HLA-identical sibling	11 (30)	37 (38.5)	
Matched unrelated	21 (57)	47 (49)	
Haplo-identical	5 (13)	12 (12.5)	
History of GVHD requiring systemic treatment, *n* (%)	21 (57)	46 (48)	0.44
Disease relapse after HSCT, *n* (%)	5 (13.5)	16 (17)	0.794
Rituximab given within the 6 months before initiation of vaccination, *n* (%)	6 (16)	0 (0)	0.000339
Systemic immunosuppression (other than rituximab) given within 3 months before initiation of vaccination^a^, *n* (%)	18 (49)	23 (24)	0.0111
*Time between HSCT and initiation of vaccination, n* (%)			0.24
≤ 12 months	11 (30)	18 (19)	
> 12 months	26 (70)	81 (78)	
*Mean cell counts over study period in PB*^b^*, n* (%)			
Lymphocytes, *n* (%)			0.00576
< 1 G/L	9 (24)	6 (6)	
≥ 1 G/L	28 (76)	90 (94)	
T cells, *n* (%)			0.144
< 1 G/L	10 (27)	15 (16)	
≥ 1 G/L	27 (73)	81 (84)	
B cells, *n* (%)			0.000643
< 0.25 G/L	20 (54)	21 (22)	
≥ 0.25 G/L	17 (46)	75 (78)	
Immunoglobulin level (g/L) at initiation of vaccination, mean ± SD	9.5 ± 5.5	9.2 ± 3.0	0.618
Third vaccine dose given	22 (59)	23 (24)	0.000189

^a^Drugs received within the 3 months preceding vaccination consisted of cyclosporine alone (*n* = 21), cyclosporine + steroids (*n* = 10), mycophenolate mofetil alone (*n* = 5) or other drug combinations (*n* = 12)

^b^Mean cell counts over study period were calculated from lymphocyte, T cell and B cell counts at time of (1) first vaccine dose (*n* = 133, 41 and 41, respectively), (2) second vaccine dose (*n* = 55, 29 and 29, respectively), (3) IgG(S-RBD) quantitation after second vaccine dose (*n* = 118, 85 and 85, respectively), (4) third vaccine dose (*n* = 40, 40 and 40, respectively) and (5) IgG(S-RBD) quantitation at 6 months (*n* = 132, 132 and 132, respectively). The corresponding mean ± SD counts for lymphocytes, T cells and B cells in both patient groups (< 1 Log or > 1 Log AU/mL at 6 months) were 1.8 ± 1.2 versus 2.2 ± 1.0 G/L (*p* = 0.0796), 1.5 ± 0.9 versus 1.7 ± 0.8 G/L (*p* = 0.191) and 0.3 ± 0.4 versus 0.4 ± 0.3 G/L (*p* = 0.0194), respectively

With a mean follow-up of 248 ± 28 days after the first vaccine dose, 4 out of 133 vaccinated recipients (3%) experienced PCR-documented SARS-CoV-2 infection within the 4–7 months following first vaccine dose. Two of those were good responders to vaccination, while the two others had IgG(S-RBD) titers below 1000 AU/mL despite a third vaccine injection (Additional file 1: Table S3). None was vaccinated before HSCT. All but one patient had mild forms of infection that could be treated in an outpatient setting. One patient who was a good responder to vaccination had a very severe and finally lethal form of COVID-19 associated with *P. Aeruginosa* pneumonia, multi-organ failure in the setting of a septic shock and EBV reactivation. Infection was preceded by a systemic steroid-refractory chronic graft-versus-host disease flare-up, occurring 5 months after the last vaccine dose and requiring a significant increase in systemic immunosuppressive therapy.

## Discussion

Kinetics of humoral response to SARS-CoV-2 mRNA vaccine has barely been studied specifically in the highly immunosuppressed population of HSCT recipients [11]. Our study allowed identification of predictive factors associated with persistence of a protective immunity at 6 months in transplanted patients, i.e., the absence of immunosuppressive medications given in the 3-to-6 months preceding vaccination (including rituximab), a lymphocyte count above 1G/L and a B-cell count above 0.25 G/L. Recipients who did not need a third vaccine dose because of their good response after 2 doses were also better protected at 6 months. Of note, our study did not show any difference in terms of IgG(S-RBD) titer at 6 months between patients vaccinated before or after 12 months post-transplant, which may be related to the fact that in the group of recipients vaccinated before 12 months post-transplant, a higher proportion of patients received an early third dose because of insufficient immune response after 2 doses (21/29 (72%) versus 24/104 (23%) for patients vaccinated after 12 months, *p* = 0.00000259).

We were able to show that persistence of a protective level of IgG(S-RBD) at 6 months highly correlated with peak antibody titers. We propose that peak antibody titer could be used as a surrogate marker for late humoral protection and guide the decision of further dose injections in case of insufficient early humoral response. In this regard, we observed a very good efficacy of an early third dose in our cohort, leading to a > 1 log increase in mean IgG(S-RBD) titers. However, similar to the general population [12], IgG(S-RBD) titers tended to decrease over time and at 6 months, only 72% of recipients retained a protective level of IgG(S-RBD), validating the need for a booster shot.

Overall, in this high-risk population, vaccination seemed to offer a good protection against COVID-19 and severe forms of the disease. Indeed, among the entire cohort of vaccinated HSCT recipients in our center, only one developed a severe form of the disease, and died, although the leading cause of death was ambiguous and complex. With a > 8 months follow-up after vaccination, we observed a 3% re-infection rate in our series, which might be in line with the cumulative incidence of SARS-CoV-2 infection close to 1% at 2 months following vaccination reported in a large series of patients with hematologic neoplasms vaccinated with the BNT162b2 mRNA vaccine [13].

The Abbott assay used in this study has a wide range of linear IgG(S-RBD) quantification, which has been shown to correlate with the level of neutralizing antibodies generated following natural infection or vaccination. The assay has been tested and validated against WHO international standard [14].

## Conclusions

In conclusion, the results of this study show that humoral response after 2 or 3 initial doses of BNT162b2 tends to decrease over time and that IgG(S-RBD) peak values below 10,370 AU/mL are associated with loss of protection at 6 months. Therefore, based on this cutoff value for peak IgG(S-RBD), additional delayed vaccine doses might be proposed to recipients showing insufficient response to vaccination. Targeting IgG(S-RBD) levels above well-defined protective thresholds in every HSCT recipient, thanks to a close monitoring of humoral response, may help improve the poor prognosis of COVID-19 in this highly immunosuppressed population. In patients with low or undetectable IgG(S-RBD) levels, alternative treatment approaches, like infusion of neutralizing monoclonal antibodies, might be discussed.

## Supplementary Information

**Additional file 1.** Supplementary Appendix.

## Data Availability

The datasets used and/or analyzed during the current study are available from the corresponding author on reasonable request.

## Abbreviations

AUC: Area under curve
Covid-19: Coronavirus disease 19
HSCT: Hematopoietic stem cell transplantation
mRNA: Messenger RNA
RBD: Receptor-binding domain
SARS-CoV-2: Severe acute respiratory syndrome coronavirus 2
