# Supplementary material for: Determinants of SARS-CoV-2 waning immunity in allogeneic hematopoietic stem cell transplant recipients
Source: J Hematol Oncol. 2022 Mar 18;15:27. doi: 10.1186/s13045-022-01250-2 (PMC8931584; doi:10.1186/s13045-022-01250-2)
Supplement: Supplementary file 1 — Additional file 1. Supplementary Appendix. [file 13045_2022_1250_MOESM1_ESM.docx]

**Supplementary Appendix**

- **Serological assays and definition of threshold values**
- **Table 1.** Safety of third BNT162b2 vaccine dose in 47 HSCT recipients (self-reported reactions)
- **Table 2.** Semi-quantitative detection of anti-N IgG following second/third vaccine dose and at 6 months
- **Table 3.** Characteristics of HSCT recipients developing SARS-CoV-2 infection despite mRNA vaccination

**Serological assays and definition of threshold values**

The threshold of 4160 AU/mL used for titers of IgG(S-RBD) is recommended by the manufacturer and currently used for surrogate measure of vaccine protection as it corresponds to a 0.95 probability of obtaining an *in-vitro* plaque reduction neutralization test.^1,2^

For the anti-N assay, index values (S/CO) reported by the instruments were used in analysis. Interpretation of the results was as follows: S/CO index >1,4 as positive; S/CO index between 0,5 and 1,4 as limit; and S/CO<0,5 as negative. The anti-S assay is an automated immunoassay that quantifies IgG(S-RBD), with 6.8 AU/mL as a limit of detection, 21 AU/mL as a minimum threshold of quantification and 40,000 AU/mL as a maximal threshold of quantification (analytical measuring interval). Samples containing IgG(S-RBD) titers higher than 40.000 AU/mL were further diluted to extend the measuring interval. All assays were performed by trained laboratory technicians according to the manufacturer standard procedures.

1. Redjoul R, Le Bouter A, Beckerich F, Fourati S, Maury S. Antibody response after second BNT162b2 dose in allogeneic HSCT recipients. Lancet Lond Engl 2021;398(10297):298–9.

2. Ebinger JE, Fert-Bober J, Printsev I, et al. Antibody responses to the BNT162b2 mRNA vaccine in individuals previously infected with SARS-CoV-2. Nat Med [Internet] 2021;Available from: https://doi.org/10.1038/s41591-021-01325-6.

**Table 1**. Safety of third BNT162b2 vaccine dose in 47 HSCT recipients (self-reported reactions)

| **Reaction and Severity** | **None** | **Mild** | **Moderate** | **Severe** |
| --- | --- | --- | --- | --- |
| *Local symptoms* |  |  |  |  |
| Pain | 19 (40.4%) | 15 (31.9%) | 13 (27.7%) | 0 |
| Redness | 38 (80.9%) | 8 (17%) | 1 (2.1%) | 0 |
| Swelling | 39 (83%) | 6 (12.8%) | 2 (4.2%) | 0 |
| *Systemic symptoms* |  |  |  |  |
| Fever | 47 (100%) | 0 | 0 | 0 |
| Chills | 45 (95.7%) | 2 (4.3%) | 0 | 0 |
| Headache | 39 (83%) | 6 (12.8%) | 2 (4.2%) | 0 |
| Fatigue | 23 (48.9%) | 19 (40.4%) | 5 (10.7%) | 0 |
| Myalgia | 29 (61.7%) | 14 (29.8%) | 4 (8.5) | 0 |
| Diarrhea | 43 (91.5%) | 4 (8.5%) | 0 | 0 |
| Vomiting | 46 (97.9%) | 1 (2.1%) | 0 | 0 |
| Erectile dysfunction | 46 (97.9%) | 0 | 1 (2.1%) | 0 |
| Facial paralysis | 46 (97.9%) | 0 | 0 | 1 (2.1%) |

**Table 2**. Semi-quantitative detection of anti-N IgG following second/third vaccine dose and at 6 months.*

|  |  | anti-N IgG detection at 6 months | | |
| --- | --- | --- | --- | --- |
|  |  | negative | limit | positive |
| anti-N IgG detection after second/third vaccine dose | negative | 110 | 8 | 0 |
|  | limit | 2 | 3 | 1 |
|  | positive | 2 | 3 | 4 |

*For the anti-N assay, index values (S/CO) reported by the instruments were used in analysis. Interpretation of the results was as follows: S/CO index >1,4 as positive; S/CO index between 0,5 and 1,4 as limit; and S/CO<0,5 as negative.

**Table 3.** Characteristics of HSCT recipients developing SARS-CoV-2 infection despite mRNA vaccination

| **Gender** | **Disease** | **Age at transplant** | **Donor type** | **GVHD requiring systemic immune suppression** | **IgG(S-RBD) titer after 2nd vaccine dose (AU/mL)** | **IgG(S-RBD) titer after 3rd vaccine dose (AU/mL)** | **Interval vaccination to infection (days)** | **SARS-CoV-2 variant** | **IgG(S-RBD) titer at time of infection (AU/mL)** | **Specific treatment of infection** | **Outcome** |
| --- | --- | --- | --- | --- | --- | --- | --- | --- | --- | --- | --- |
| M | Myeloma | 39 | Pheno-id 10/10 | no | 46394 | _ | 186 | Delta | nd | none | alive |
| M | Primary myelofibrosis | 57 | Pheno-id 10/10 | yes | 21 | 278 | 137 | Alfa | nd | none | alive |
| M | Acute myeloid leukemia | 64 | Geno-id | yes | 7 | 7 | 188 | Delta | nd | casirivimab/  imdevimab | alive |
| M | Chronic lymphocytic leukemia | 43 | Haplo-id | yes | 19025 | _ | 217 | Delta | 1719 | Dexamethasone | dead (chronic GVHD flare-up, P. aeruginosa pneumonia, EBV reactivation) |
